# Toward personalized persuasive social robots for behavior change in healthcare: a conceptual framework

**DOI:** 10.3389/frobt.2026.1760008

**Published:** 2026-02-24

**Authors:** Elie Maalouly, Alessandra Rossi, Silvia Rossi

**Affiliations:** 1 Department of Neuroscience and Reproductive and Odontostomatological Sciences, University of Naples Federico II, Naples, Italy; 2 Department of Electrical Engineering and Information Technologies, University of Naples Federico II, Naples, Italy

**Keywords:** behavior change, conversation system, framework, human-robot interaction, personalization, persuasion

## Abstract

This paper presents a conceptual framework for the design of personalized persuasive conversational agents to support positive behavior change. This paper leverages key theoretical models to understand the determinants of behavior change and explores how these models can inform the design of personalized conversational agents to enhance their effectiveness in healthcare interventions. The role of personalization in dialogue-based intervention is discussed, emphasizing the importance of adaptation to individual characteristics, preferences, and contexts. The potential of persuasive language generation is also examined, highlighting its ability to create more engaging and impactful behavior change strategies. Finally, the paper proposes a layered framework that explicitly links behavioral models, user personalization, and persuasive language generation, and discusses future research directions for integrating this framework in social robots’ interventions for behavior change in healthcare.

## 1 Introduction

Consistent engagement in beneficial behaviors is associated with better mental and physical health (White et al., 2024; Zhang et al., 2024), improved performance at work, in social life, and education (Martín-Rodríguez et al., 2024; Han et al., 2025), and more sustainable decision-making (Trudel, 2018; Kim and Lee, 2023). To address social challenges, numerous models of behavior change have been developed to explain and predict why people act as they do. While robotic systems have been applied to behavior change (Robinson et al., 2019), evidence shows that interventions work best when grounded in appropriate theoretical models (Rhodes et al., 1997).

Understanding what drives human behavior is essential, but not sufficient. Behavior change is an internal process where people need to “want” to change their behavior. Since desired behaviors cannot be imposed, persuasion becomes a key strategy to foster voluntary change. In healthcare, persuasive interventions use technology to encourage healthier behaviors, such as increasing activity, eating well, quitting smoking, or managing stress (AlSlaity et al., 2022; Oyebode and Orji, 2022). Since the early 2000s, most persuasive health technologies have used a “one-size-fits-all” approach (Fogg, 2002; Busch et al., 2015), overlooking differences among users. Yet, individuals respond differently to persuasion due to factors like personality, demographics, and environment (Alslaity and Tran, 2021; Abdullahi et al., 2019). Personalization, the process of tailoring content, features, and interactions to each user’s traits and goals, aims to address this variability (Fan and Poole, 2006).

The robotic systems discussed in this paper follow Fong et al. (2003)’s definition of socially interactive robots: embodied agents capable of perceiving and expressing emotion, communicating through dialogue, using natural cues (e.g., gaze, gestures), forming social relationships, and displaying personality. A Conversational Agent (CA) is a dialogue system that understands and generates natural language for text or voice interaction (Allouch et al., 2021). Social robots typically include a CA but extend it with a body, sensors, and social presence in the physical world. The two terms are distinct and are thus not used interchangeably in this paper. Recent surveys have reviewed conversational agents (Pereira and Díaz, 2019), persuasive technologies (Adaji and Adisa, 2022), and social robots for behavior change (Nocentini et al., 2019), focusing mainly on functionalities, personalization, or outcomes. Yet, these studies often treat behavior change theories, personalization, and persuasive language generation as distinct dimensions, lacking a unified framework that links behavior models to user modeling and personalized persuasion strategies in healthcare CAs and social robots. Consequently, designers still lack integrated guidance for translating theoretical constructs into concrete personalization and persuasive dialogue choices. In this work, we contribute a conceptual framework that systematically connects behavior change theories, user personalization mechanisms, and persuasive language generation within conversational agents and social robots, with a specific focus on healthcare applications.

In this conceptual paper, we will focus on the conversational aspect of robotic systems for behavior change. Conversational interaction is particularly suitable for behavior change because it enables real-time assessment of user readiness (Azios et al., 2020), rapport-building through dialogue (Angelo, 2015), and adaptive personalization of persuasive strategies (Shree Smeka et al., 2025). We will first examine theoretical models for behavior change, then discuss how these models can be used to create personalized persuasive Conversational Agents. We will also explore the potential of personalization in persuasive language generation and how it can be used to create more effective behavior change interventions. Finally, we will discuss future directions for research in this area that can guide researchers in the development of CAs for behavioral changes.

## 2 Theoretical models for behavior change

Successful health behavior interventions can be developed and assessed only with the knowledge of the factors affecting people’s behaviors (Glanz et al., 1990). Behavioral models and theories try to clarify why individuals act in certain ways, by aiming to pinpoint the variables that influence the choices to partake in or refrain from particular behaviors (Rhodes et al., 1997). The most widely prevalent and used models are the Health Belief Model (Becker, 1974), the Theory of Reasoned Action (Ajzen, 1980), the Social Cognitive Theory (Bandura, 2002), and the Transtheoretical Stage-of-Change Model (Prochaska and Diclemente, 1986).

### 2.1 Health belief model

The Health Belief Model (HBM) (Becker, 1974) includes two primary factors that impact an individual’s probability of implementing a preventative measure that is advised to mitigate an existing health hazard. The patient must first believe that they are personally susceptible to the illness. The illness must also be regarded as having grave or dangerous repercussions. The second requirement is that the person must think that the suggested course of action would successfully lessen the perceived health hazard and that there will be more advantages from following the preventative activity than perceived costs.

This model succeeds by targeting the threats and benefits of certain behaviors, which make it particularly suitable for health-related behavior. However, it can be regarded as reductionist as it ignores social and environmental factors (Davidhizar, 1983).

### 2.2 Theory of reasoned action

According to the Theory of Reasoned Action (TRA), behavioral intentions are influenced by attitudes, subjective norms, and perceived behavioral control, which are all connected to actual behavior (Ajzen, 1980; Hill et al., 1977). The intention of the individual to carry out an action is the main factor that determines behavior. This intention is seen as the result of two factors: the individual’s attitude toward engaging in the behavior (e.g., beliefs about the benefits and costs of engaging in the behavior), and the individual’s perception of the pressure from society, or norms, to engage in the behavior. Even though this model excels in social intention prediction, it still assumes rationality (fails at predicting habits and impulses) (Rossmann, 2020).

### 2.3 Social cognitive theory

The Social Cognitive Theory (SCT) highlights two main factors that affect how likely someone is to take preventative action (Bandura, 2002). A person must first think that the advantages of engaging in the conduct outweigh the disadvantages, similar to the HBM and the TRA. The second, and possibly most crucial requirement, is that the individual must feel capable of carrying out a certain preventative action. This is known as self-efficacy (Bandura, 2002; 1977). The individual must think that they possess the knowledge and talents required to carry out the conduct under a range of conditions and in the face of diverse challenges. This model, however, underemphasizes biological and genetic factors in favor of learned environment (McLeod, 2011).

### 2.4 The transtheoretical stage-of-change model

The Transtheoretical Model (TTM) was created to outline the phases that people usually go through when altering their behavior (Prochaska and Diclemente, 1986; Prochaska, 1992; Prochaska et al., 1997). According to the TTM, people’s readiness to change their behavior varies in stages that differ qualitatively in terms of the concepts and procedures that lead individuals toward specific behaviors. As a result, the model states that interventions should be given to individuals based on where they are in the behavior change stage. The model depicts the following stages:

1. Precontemplation: the person is not planning nor even considering adopting a certain behavior.
2. Contemplation: the person intends to adopt a behavior, but has not yet started doing so.
3. Preparation: the person is intending to change in the near future and is making an effort to do so.
4. Action: the person has maintained the new behavior over a period shorter than 6 months.
5. Maintenance: the person has maintained the new behavior for over 6 months.

Although this model is unique in its stage-matching, this also serves as a point of criticism as the stages might seem arbitrary and progression through stages might not necessarily correspond to a change in behavior (Brug, 2004).

### 2.5 Dynamic and longitudinal adaptations of behavioral models

Behavior change models, originally static, can be extended for dynamic, longitudinal interactions via CAs’ conversational memory, real-time sensing, and NLP to track progress across sessions. For TTM, repeated dialogues can assess stage transitions using readiness questions, with relapse prompting stage regression (Zhang et al., 2020). SCT self-efficacy can be built via logged mastery experiences (“You’ve succeeded 3 times! Try longer next?“). HBM can update susceptibility from health data and TRA can update norms from social probes. Longitudinal studies confirm sustained engagement via ecological momentary assessments for relapse detection, though ethical data privacy remains key (Stanojevic et al., 2022).

### 2.6 Selecting a behavioral model for intervention design

Selecting the appropriate behavioral model remains critical, as theory-grounded interventions significantly outperform atheoretical approaches (Rhodes et al., 1997). Table 1 shows examples linking healthcare scenarios to optimal behavioral models:

**TABLE 1:** A mapping of healthcare scenarios to optimal behavioral model choice.

Healthcare scenario	Model	Example studies
Physical activity increase	SCT	Lubans et al. (2016), Barrett et al. (2018), Ek et al. (2018), Murawski et al. (2018), Demetriou and Bachner (2019)
Improving diet and nutrition	HBM, TTM, SCT	Nakamura et al. (2017), Patel et al. (2019), Bestle et al. (2020), Abildsnes et al. (2017)
Cardiovascular disease medication adherence	HBM, SCT	Kamal et al. (2016)
Hygiene improvement	SCT	Delea et al. (2019)
Promote preventative measures for COVID-19	HBM, SCT	Conroy (2022)
Treatment of internet gaming disorder	TTM	Pakpour et al. (2022)
Intervention for weight management	SCT, HBM, TTM	Rashid et al. (2022)
Oral health habit promotion	TTM	Wang et al. (2022)

All the models presented here can be modified to investigate and address more complex behaviors as well. Combining theories is a popular practice among researchers and practitioners, and it can be beneficial when done methodically and carefully. The development of a logic model is a crucial tool for achieving this (Bartholomew et al., 2015).

## 3 Behavior change through conversational agents

Once the most appropriate behavioral theory is chosen, or a mix of theories is constructed, how can a robotic application be designed to support the change in people’s behavior?

A popular and effective way is through the design of CAs. Our focus in this paper is on interactive conversation as it can be a powerful tool to be used to provide information, answer questions, and even persuade users to change their behavior (Pereira and Díaz, 2019). Having the skills to communicate effectively and clearly can have a profound effect on someone, causing departure or adoption of certain health-related behaviors. In the context of healthcare-related behavior change, CAs interact with patients to monitor, guide, motivate, or prevent specific behaviors (Martinengo et al., 2022). Pereira and Díaz (2019) mapped this domain using a triplet < illness, competences, technical enablers > , where competences represent desired patient behaviors and enablers denote chatbot capabilities that enhance engagement. Among these, personalization emerged as a key enabler for effective design. Identifying such enablers helps researchers focus on the most promising strategies (such as personalization) to develop more impactful CAs for health-related behavior change.

## 4 Personalization

Personalization generally refers to addressing patients’ unique needs to increase satisfaction and treatment engagement (Pereira and Díaz, 2019). It facilitates adaptation to patients’ medical backgrounds and conditions, benefiting both physical and nutritional health. For example, personalized systems support diabetes management (Cheng et al., 2018), offer individualized diet guidance (Huang et al., 2018; Gabrielli et al., 2018), and enable tailored health screening, guidance, and motivation (Cameron et al., 2017; Callejas et al., 2014; Fadhil and Gabrielli, 2017; Ly et al., 2017).

Kocaballi et al. (2019) proposed a framework to characterize personalization along three dimensions:

1. What is personalized? (i.e., content, user interface, delivery channel, and functionality)
2. For whom is it personalized? (individual or group)
3. How automated is the personalization? (the method of gathering user modeling data)

The process relies on user models capturing characteristics, preferences, and needs to deliver adaptive content and services. Based on automation level, personalization may be implicit, where data are automatically inferred from user behavior, or explicit, where users actively provide the necessary information.

Personalization evolves temporally through hybrid implicit and explicit updates. Initial explicit data (questionnaires) shifts to implicit learning from interaction histories and behavioral logs, enabling dynamic adaptation. Yet, risks include privacy concerns from longitudinal data, biased model drift, over-reliance eroding empathy, and hallucinations yielding unsafe advice (Laymouna et al., 2024; Bickmore et al., 2010) According to Kocaballi et al. (2019), personalization can target various aspects, including content, user interface, delivery channel, and functionality. In practice, nearly any element can be personalized, as the concept imposes no strict limits. A frequently overlooked aspect in behavior change CAs is the personalization of Persuasive Language Generation (Iyer and Sycara, 2019). To grasp its potential, it is essential to first understand the role of persuasion and persuasive language generation.

## 5 Persuasive language generation

According to Iyer and Sycara (2019), persuasion is the process by which one party, known as the persuader, tries to get the other party, known as the persuadee, to believe or disbelieve something or to do something. A persuasive Natural Language Generation (NLG) Artificial Intelligence (AI) generates messages aimed at convincing a user (the persuadee) to accept an argument. Recent NLG advances show pretrained language models achieve state-of-the-art results across NLP tasks (Wang et al., 2023), enabling human-like text that can be optimized for persuasion.

In contrast to an expression of sentiment, persuasion aims to alter the persuadee’s mental state. According to modern psychology and communication science (Park et al., 2015; Hunter et al., 2019), persuasion requires the persuader to be acting on purpose, or performing a persuasive act.

Psychological research has identified several persuasion strategies that frequently occur in human interactions. Cialdini (2007) highlighted six main principles of persuasion, including reciprocation, where people feel a sense of obligation to return favors; consistency, which reflects the human desire to act in accordance with one’s values and prior commitments; social proof, the tendency to follow the behavior of others; liking, where individuals are more easily persuaded by those they have positive feelings toward; authority, where messages from experts or credible sources are more convincing; and scarcity, the perception that something limited in availability is more valuable or desirable. These principles of persuasion can be embedded into formal “theories of persuasion” such as the Theory of Cognitive Dissonance (O’Keefe, 1990), Language Expectancy Theory (LET) (Burgoon and Miller, 1985), and Balance Theory (Heider, 2013). For an in-depth review of the theories of persuasion, please refer to Cameron (2009).

These principles must navigate ethical boundaries between legitimate influence and manipulation: ethical persuasion respects autonomy via transparency and evidence-based strategies, while manipulation coerces through deception or exploitation (Erren et al., 2023). In healthcare CAs, this translates to NLG grounded in behavior change techniques (Martinengo et al., 2022).

As computing permeates every aspect of life, applying computer-based solutions to persuasion is becoming a goal. Human-computer interaction research advances, such as the seminal work by Fogg (1998), have given rise to persuasive technologies, which focus on meeting the demand for tools that support people in changing their behavior, especially when it comes to healthcare and lifestyle decisions. Current persuasion tools do not offer explicit argumentation that takes into account arguments and counterarguments. However, an argument-based strategy may be quite helpful for specific behavior modification objectives, especially if the person is deficient in facts or harboring misconceptions about the subject.

## 6 Proposed framework

We believe that the integration of persuasive language generation, personalization, and behavioral models presents a promising avenue for advancing behavior change interventions. We present the following layered framework as a concrete way to bridge the segmentation in the field.

The first step consists of choosing an underlying behavioral model. Behavioral models provide structure that will guide the dialogue generation through the setting of goals to be achieved through persuasive conversation. The HBM, for example, would provide a structure that would guide the conversation toward making the patient understand the health risks of their current action and how a change in behavior would lessen this perceived risk. The TTM would require evaluating on which stage the patient is currently through a short questionnaire and aiming the conversation to trigger the patient’s transition into the next stage. Models can adapt dynamically, for example, TTM stages may change via conversation history and SCT self-efficacy can be updated longitudinally via performance logs.

Once an appropriate behavioral model is chosen, the next step is to determine the personalization strategy. This will involve building a user model (implicitly through interaction history or explicitly by answering a short questionnaire). What is included in the user model will ultimately guide the persuasion strategy and the dialogue generation. The user model will inform what kind of data (demographics, occupation, education level, personality, decision-making style, moral values, etc.) will be used for personalizing the persuasion strategy. The context and scenario will also play a role in informing the persuasion strategy.

**FIGURE 1:**
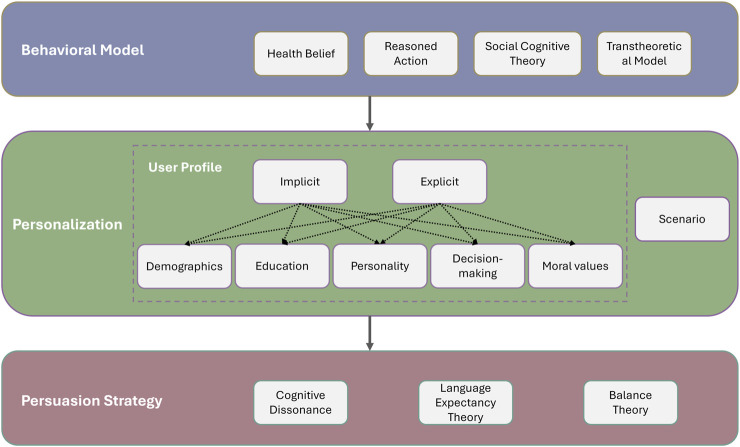
Framework for integrating behavioral models, personalization and persuasive language generation.

Finally, persuasive language generation delivers the message. Once a model-informed goal and a personalization strategy are chosen, persuasive NLG produces the actual utterance. This involves choosing rhetorical style and argument form in line with theories like Cognitive Dissonance (to reduce conflicting beliefs), Language Expectancy Theory (to remain within culturally appropriate norms), or Balance Theory (to strengthen trust in the agent). This presented framework is illustrated in Figure 1.

As an illustrative example, let us suppose the CA is designed with TTM as a backbone. A user in the contemplation stage may express doubt, “I know exercise is good, but I do not think I have time for it”. The agent, guided by the model, targets self-efficacy as the key construct to support. Personalization draws on the user’s context (limited free time, preference for short activities). Persuasive language generation then produces a supportive utterance that both validates the barrier and offers a small, feasible suggestion, “Even a 10-min walk after lunch can make a real difference; many people with busy schedules find that manageable”.

This integration illustrates how behavioral models, personalization, and persuasive language can complement one another: the model defines the “what”, personalization chooses the “how”, and persuasive language determines the “how it sounds”.

As another example, let’s consider a 45 year old teacher trying to quit smoking says, “I know I should quit, but stress from work makes it impossible.” The CA with the SCT as a behavioral model targets self-efficacy as the key barrier. Personalization uses the user’s profession and seasonal stress patterns (from profile and interaction history). Persuasive NLG responds: “Many teachers face grading stress too. Your organized nature that helps with lesson plans can work here: a 2 min deep breathing exercise between papers worked for colleagues. Your last attempt lasted 3 days; let’s beat that tomorrow?” While this paper primarily focused on personalizing persuasive conversations for robotic systems, effective behavior change requires broader consideration. Beyond dialogue, robots can leverage adaptive behavioral models, cognitive architectures, and empathetic strategies to influence behavior. By interpreting users’ emotions, intentions, and habits through cues like gaze, posture, and tone, social robots can adapt responses in real time to encourage desired actions (Chidambaram et al., 2012). Assistive robots, for instance, can promote healthy routines, issue reminders, and motivate social or rehabilitative engagement (Fasola and Mataric, 2013; Fiorini et al., 2016). Integrating empathy and cultural adaptation enhances trust and relevance, helping robots act as supportive companions that foster long-term behavioral change and inclusion (Nocentini et al., 2019; Chen et al., 2014).

## 7 Ethical considerations and safeguards

It is also important to stress the ethical challenges that arise when using personalized persuasive CAs. While this framework promises enhanced effectiveness through integrated persuasion, it raises ethical concerns where personalized NLG risks manipulation, such as covertly exploiting a depressed user’s vulnerabilities to recommend unproven supplements (“Only this pill can save your health”), delivering culturally biased advice (“Your background makes change impossible; just try harder”), or failing to escalate suicide risk signals with generic positivity (“Think happy thoughts”). To mitigate these, design principles include full transparency about AI nature and persuasion goals, user autonomy via opt-out options and human escalation for high-risk cases, rigorous fact-checking and bias audits of NLG outputs.

Ethical considerations must be addressed to ensure that these systems are designed and deployed responsibly. Fogg’s work on persuasive technologies (Fogg, 1998) provides a foundation for ethical design principles that can guide the development of personalized persuasive CAs.

## 8 Conclusion

Due to the segmentation of the research in this area, we can see that there is a lack of, and therefore a need for, interdisciplinary collaboration. The integration of behavioral models, personalization, and persuasive language generation requires expertise from various fields, including psychology, artificial intelligence, and healthcare. Collaborative efforts could lead to the development of more robust frameworks that leverage the strengths of each discipline. Partnerships with healthcare providers could facilitate the deployment of these systems in clinical settings.

While our framework advances personalized persuasive CAs for behavior change, long-term CA interventions face challenges in engagement (waning due to repetitiveness) (Bickmore et al., 2010), adherence (barriers like priorities, motivational decay) (Middleton et al., 2013), and high drop-out (Meyerowitz-Katz et al., 2020). Personalization boosts initial retention but requires dynamic variability for sustained use. Our framework counters these via model-guided, adaptive persuasion, yet demands longitudinal validation. Despite the progress made, significant gaps remain. Future research must focus on developing unified frameworks that combine these elements while addressing ethical challenges. Collaboration across disciplines, including psychology, artificial intelligence, and healthcare, will be essential to significantly advance this field.

## Data Availability

The original contributions presented in the study are included in the article/supplementary material, further inquiries can be directed to the corresponding author.
